# Homology Modeling Informs Ligand Discovery for the Glutamine Transporter ASCT2

**DOI:** 10.3389/fchem.2018.00279

**Published:** 2018-07-24

**Authors:** Rachel-Ann A. Garibsingh, Nicholas J. Otte, Elias Ndaru, Claire Colas, Christof Grewer, Jeff Holst, Avner Schlessinger

**Affiliations:** ^1^Department of Pharmacological Sciences, Icahn School of Medicine at Mount Sinai, New York, NY, United States; ^2^Origins of Cancer Program, Centenary Institute, University of Sydney, Sydney, NSW, Australia; ^3^Sydney Medical School, University of Sydney, Sydney, NSW, Australia; ^4^Department of Chemistry, Binghamton University, Binghamton, NY, United States

**Keywords:** homology modeling, SLC1A5, glutamine transporter, solute carrier transporter, structure prediction, cancer metabolism, structure-based ligand discovery

## Abstract

The Alanine-Serine-Cysteine transporter (SLC1A5, ASCT2), is a neutral amino acid exchanger involved in the intracellular homeostasis of amino acids in peripheral tissues. Given its role in supplying glutamine to rapidly proliferating cancer cells in several tumor types such as triple-negative breast cancer and melanoma, ASCT2 has been identified as a key drug target. Here we use a range of computational methods, including homology modeling and ligand docking, in combination with cell-based assays, to develop hypotheses for structure-function relationships in ASCT2. We perform a phylogenetic analysis of the SLC1 family and its prokaryotic homologs to develop a useful multiple sequence alignment for this protein family. We then generate homology models of ASCT2 in two different conformations, based on the human EAAT1 structures. Using ligand enrichment calculations, the ASCT2 models are then compared to crystal structures of various homologs for their utility in discovering ASCT2 inhibitors. We use virtual screening, cellular uptake and electrophysiology experiments to identify a non-amino acid ASCT2 inhibitor that is predicted to interact with the ASCT2 substrate binding site. Our results provide insights into the structural basis of substrate specificity in the SLC1 family, as well as a framework for the design of future selective and potent ASCT2 inhibitors as cancer therapeutics.

## Introduction

The human SLC1 family is comprised of seven sodium-dependent amino acid transporters, including five excitatory amino acid transporters (EAAT1-5) and two alanine-serine-cysteine transporters (ASCT1 and ASCT2) (Kanai et al., 2013). The EAATs are primarily expressed in the central nervous system (CNS), where they mediate the uptake involved in the termination of glutamate neurotransmission (Danbolt, 2001; Vandenberg and Ryan, 2013). The ASCTs facilitate exchange of neutral amino acids in peripheral tissues such as the kidney, intestine, and skin and/or the CNS, and they are responsible for maintaining intracellular homeostasis of amino acids (Bröer et al., 1999; Kanai et al., 2013). Genetic variations in SLC1 members can lead to a variety of diseases and disorders. For example, mutations in EAAT1 (SLC1A3) that reduce its activity, can cause neurodegenerative disorders such as stroke (Chao et al., 2010) and ataxia (Winter et al., 2012; Choi et al., 2017). Conversely, the upregulation of ASCT2 occurs in many cancers such as triple-negative breast cancer (Kim et al., 2013; van Geldermalsen et al., 2016), melanoma (Wang et al., 2014) and prostate cancer (Wang et al., 2013), and can correlate with poor prognosis, shown in studies of hepatocellular carcinoma (Namikawa et al., 2014; Sun et al., 2016) and non-small cell lung cancer (Shimizu et al., 2014; Yazawa et al., 2015).

One of the hallmarks of cancer is the alteration of tumor cell metabolism to support rapid growth and proliferation (DeBerardinis et al., 2007; Schulze and Harris, 2012; Altman et al., 2016). This increased need for carbon and nitrogen sources can result in increased reliance of cells on glutamine, in a phenomenon commonly known as “glutamine addiction” (Wise and Thompson, 2010; Bhutia et al., 2015; Altman et al., 2016). Glutamine is imported into tumor cells primarily by ASCT2 (Wasa et al., 1996; Collins et al., 1998; Fuchs et al., 2007; Scalise et al., 2017), where it is used for nucleotide synthesis (Lane and Fan, 2015) and to replenish TCA cycle intermediates (Albers et al., 2012; Schulze and Harris, 2012; Pochini et al., 2014). This glutamine can also contribute to mTORC1 activation and subsequent proliferation by being exchanged for leucine via LAT1 (Nicklin et al., 2009; Pochini et al., 2014; Wang et al., 2014).

Previous studies have shown that knock down of ASCT2 results in tumor growth attenuation *in vitro* (Nicklin et al., 2009; Wang et al., 2014, 2015; van Geldermalsen et al., 2016; Schulte et al., 2018) and *in vivo* (Wang et al., 2015; van Geldermalsen et al., 2016), and the viability of ASCT2 knockout mice suggests that pharmacological targeting of ASCT2 may not affect normal cells (Nakaya et al., 2014; Masle-Farquhar et al., 2017). Indeed, a recent study showed that pharmacological inhibition of ASCT2 reduced cancer cell growth and proliferation *in vitro and in vivo* (Schulte et al., 2018). This information taken together purports that ASCT2 is a significant drug target. To develop potent and specific compounds for ASCT2, a detailed understanding of its substrate specificity and binding site properties is needed.

Experimentally determined structures or well-made homology models, can be extremely powerful to uncover novel chemical scaffolds when combined with structure-based virtual screening (Colas et al., 2015). Currently, there are no experimentally solved structures of ASCT2 and ASCT1. Most of the knowledge about the human SLC1 family structure and molecular mechanism came from the study of their prokaryotic homologs, the aspartate transporters, Glt_Ph_ from *Pyrococcus horikoshii* (Yernool et al., 2004) and Glt_Tk_ from *Thermococcus* kodakarensis (Guskov et al., 2016) that share a 77% sequence identity (Guskov et al., 2016). Similar to ASCT2, these transporters couple substrate transport with the cotransport of three Na^+^ ions (Groeneveld and Slotboom, 2010; Kanai et al., 2013). Moreover, they have eight transmembrane helices (TMs) and share sequence identity of ~30% and a conserved binding site with the human members of the SLC1 family (Yernool et al., 2004; Albers et al., 2012; Scopelliti et al., 2013; Colas et al., 2015; Canul-Tec et al., 2017).

The Glt_Ph_ structures have been determined in multiple conformations of the transport cycle (Verdon et al., 2014) and with a variety of substrates and inhibitors (Yernool et al., 2004; Boudker et al., 2007; Verdon et al., 2014; Scopelliti et al., 2018). These structures, combined with characterizations using other biophysical methods (e.g., smFRET), have revealed that the transporter exists as a trimer and transports its substrates using an “elevator” transport mechanism (Reyes et al., 2009, 2013; Akyuz et al., 2013) (**Figure 3**). In brief, in the elevator mechanism, the transporter has a mobile transport domain that binds the substrate and traverses the membrane, and a static scaffold domain that mediates oligomerization (Hirschi et al., 2017). Recently, the human EAAT1 atomic structure was solved in various conformations, bound to aspartate or competitive inhibitor (TBOA), as well as with an allosteric inhibitor (Canul-Tec et al., 2017). This structure was highly similar to that of Glt_Ph_, confirming the relevance of Glt_Ph_ for studying the human family. Furthermore, these structures suggest that the other human SLC1 members including ASCT2 operate via a similar transport mechanism (Boudker et al., 2007; Reyes et al., 2009; Canul-Tec et al., 2017).

Multiple models of human SLC1 members have been constructed based on the Glt_Ph_ structure and tested experimentally using a variety of biochemical and biophysical methods (Yernool et al., 2004; Albers et al., 2012; Scopelliti et al., 2013; Colas et al., 2015; Console et al., 2015; Canul-Tec et al., 2017). These studies identified key binding site residues that explain the differential charge specificity among these proteins (Scopelliti et al., 2013, 2018; Colas et al., 2015; Canul-Tec et al., 2017; Singh et al., 2017). We have previously built models of ASCT2 based on the outward-occluded and outward-open conformations of Glt_Ph_ and used these models to identify and refine multiple ASCT2 ligands, including inhibitors and substrates (Colas et al., 2015; Singh et al., 2017). Interestingly, a recently solved structure of a Glt_Ph_ variant that was engineered to bind some ASCT2 substrates, provided a model to understand ASCT2-ligand interactions (Scopelliti et al., 2018). This structure also revealed a binding site conformation similar to that of our model, confirming our modeling approach.

Here, we model ASCT2 structure in two conformations based on the newly solved EAAT1 structures and evaluate the relevance of these models for drug discovery. Particularly, we first analyze phylogenetic relationships among human SLC1 family members and their prokaryotic homologs, to inform the homology modeling of this family. We then generate models of ASCT2 and perform a comparative analysis to the engineered Glt_Ph_ variant crystal structures, to assess the utility of the various proteins in ligand discovery. We also describe the discovery of a unique ASCT2 inhibitor from virtual screening and predict its mode of interaction with ASCT2. Our results provide insights into the structural basis of substrate specificity in the SLC1 family and provides a framework for the design of ASCT2 inhibitors with improved selectivity and potency.

## Materials and methods

### Phylogenetic tree

To build the phylogenetic tree of the SLC1 members, FASTA sequences of each family member were retrieved from Uniprot (Uniprot Consortium, 2018) and aligned with MUltiple Sequence Comparison by Log-Expectation (MUSCLE) (Edgar, 2004) and Promals3D (Pei and Grishin, 2014). This multiple sequence alignment (MSA) in FASTA format was used as input for Simple Phylogeny, which is a web server that performs phylogenetic analysis on multiple sequence alignments (Larkin et al., 2007; McWilliam et al., 2013). The Newick tree format was selected as the output, for its ability to be viewed with multiple tree viewing programs. Distance correction was set to on, to avoid branch stretching for more divergent sequences. Exclude gaps was set to on, to remove gaps in the MSA and use only the positions where information could be included from all sequences. The Neighbor-joining algorithm was used to construct the tree from the distance matrix. The final tree was visualized with FigTree (Rambaut, 2012), a graphical program for viewing phylogenetic trees.

### Homology modeling

ASCT2 was modeled based on X-ray structures of human EAAT1 in the outward-occluded and outward-open conformations (PDB codes 5LLM and 5MJU, respectively) (Canul-Tec et al., 2017). The initial ASCT2-EAAT1 alignment was derived from the MSA of human SLC1 family members and prokaryotic homologs, which was subsequently refined based on visual analysis of (i) a pairwise ASCT2-EAAT1 alignment generated by Promals3D (Pei and Grishin, 2014) under default parameters (ii) previously published alignments of the SLC1 family (Yernool et al., 2004; Canul-Tec et al., 2017); (iii) preliminary homology models generated based on the various alignments. We omitted from modeling, large variable loop regions that are distant from the substrate binding site and are unlikely to affect ASCT2-ligand interactions (Colas et al., 2015). These regions included the loops between transmembrane (TM) region 3 and TM4a and between TM4b and TM4c. For each conformation and ASCT2-EAAT1 alignment, 100 initial models were constructed with MODELLER-v9.11 (Webb and Sali, 2017). The initial models were refined by iteratively modeling the sidechains of D464 and M387 on a fixed backbone with PyMOL^1^ and visually assessing the generated models. The models were constructed with nonprotein elements, including the sodium ions, as well as the ligands aspartate or TBF-TBOA and UCPH_101_ based on their coordinates in the template structures. The models were then evaluated and ranked by their Z-DOPE score, a normalized atomic distance dependent statistical potential based on known protein structures (Shen and Sali, 2006). The top-scoring models had Z-DOPE scores of −0.517 (outward-occluded) and −0.313 (outward-open), respectively. These scores are significantly better than previously published models of ASCT2 using Glt_Ph_ structures as modeling templates, indicating that they are suitable for further investigation. The 15 top-scoring models for each conformation, were subjected to enrichment analysis with ligand docking. All models are available for direct download through www.schlessingerlab.org/data.

### Ligand docking and enrichment

We evaluated the ability of the models to distinguish known ASCT2 ligands from decoys using ligand enrichment calculations (Huang et al., 2006; Fan et al., 2009). For the outward-open model, 26 known ASCT2 ligands were obtained from the literature (Esslinger et al., 2005; Albers et al., 2012; Oppedisano et al., 2012; Colas et al., 2015; Schulte et al., 2015, 2016; Singh et al., 2017) and ChEMBL (Gaulton et al., 2017), and 1304 decoys were generated based on these ligands using the DUD-E server (Mysinger et al., 2012). For the occluded models, we removed 7 ASCT2 ligands that are large and unlikely to fit into the substrate binding site. Thus, 19 known ligands were used to generate 937 decoys with DUD-E for this conformation. Docking was performed using OpenEye FRED (McGann, 2011). We chose to use OpenEye FRED based on the following criteria: (i) docking against ASCT2 models obtained high enrichment scores as compared to scores obtained with other methods (not shown) (ii) the program is available through an academic license; (iii) its speed and ease of use, a feature that enables us to perform docking against multiple models, which has been shown to increase prediction accuracy and ligand enrichment (Fan et al., 2009); and (iv) OpenEye FRED has shown its usefulness in ligand discovery campaigns targeting models of transporters with chemically related substrates, such as the L-type amino acid transporter LAT1 (Zur et al., 2016) and the oligopeptide transporter PepT1 (Colas et al., 2017). The binding site was prepared using the MAKE_RECEPTOR utility of FRED with (S351 and N471 ASCT2 outward-occluded constraints and S351 and S353 ASCT2 outward-open constraints) and ligand conformations were prepared with the OMEGA utility. Finally, the area under the curve (AUC) and the logarithmic scale AUC (logAUC) of the enrichment plots were calculated (Fan et al., 2009).

### Virtual compound screening

We used OpenEye FRED (McGann, 2011) as described above to screen the ZINC15 “Available Now Lead-Like” library against our outward-open ASCT2 model based on Glt_Ph_ (8.7 x 10^6^ compounds). We used constraints on the backbone nitrogen of S353 and the carboxy group of the side chain of D464. We visually inspected the 200 top-scoring compounds of the screen and prioritized compounds predicted to interact with the binding site through conserved hydrogen bonds with important residues between ASCT2 and Glt_Ph_ (**Figure 2**). Additionally, of those compounds, we selected 13 compounds that included potentially new chemotypes as ASCT2 ligands and also docked in both pocket A (PA) and pocket B (PB) (Supplementary Table 1).

### Pocket volume measurement

POVME3 (Wagner et al., 2017) (Pocket Volume MEasurer) was used to calculate binding site volumes. The coordinates of the binding site were estimated using AutoDock Vina plugin (Trott and Olson, 2010) in PyMOL. These coordinates were used as input for POVME3.

### SK-MEL-28 cell culture

Human melanoma cancer cell line SK-MEL-28 identity was confirmed by STR profiling in 2018 (Cell Bank, Sydney, Australia). Cells were routinely tested as free from mycoplasma using a PCR-based detection method. SK-MEL-28 cells were grown in DMEM medium containing 4 mM L-glutamine and 20 mM HEPES (Life Technologies), supplemented with 10% (v/v) fetal bovine serum (FBS; HyClone) and 1× penicillin-streptomycin solution (Life Technologies). Cells were passaged every 2–3 days and were maintained in a fully humidified atmosphere containing 5% CO_2_ at 37°C. Inhibitors were resuspended in DMSO to 20 mM, before being diluted to their final concentrations in uptake media. L-γ-glutamyl-p-nitroanilide (GPNA) was resuspended in water prior to dilution in uptake media to final concentrations.

### Glutamine uptake assay

SK-MEL-28 cells (1 × 10^5^/well) were incubated with [^3^H]-L-glutamine (400 nM; PerkinElmer) in RPMI media (Life Technologies) without L-glutamine for 15 min at 37°C in the presence or absence (Control; vehicle) of each inhibitor as previously published (van Geldermalsen et al., 2016). Cells were collected and transferred to filter paper using a 96-well plate harvester (Wallac PerkinElmer), dried, exposed to scintillation fluid and counts measured using a liquid scintillation counter as previously published (MicroBeta^2^ Counter, PerkinElmer) (Wang et al., 2014).

### Electrophysiology

Experiments were performed as described in detail previously (Albers et al. 2012). In brief, compound **10** was dissolved in DMSO and diluted to a maximum concentration of 1 mM (5% final DMSO, DMSO at this concentration did not cause any notable currents), before application to HEK293 cells expressing rat ASCT2 and suspended from a current recording electrode. External buffer used to dissolve the compound was; 140 mM NaCl, 2 mM MgCl_2_, 2 mM CaCl_2_, 10 mM HEPES at pH 7.40. Intracellular buffer contained 130 mM NaSCN, 2 mM MgCl_2_, 10 mM EGTA, 10 mM HEPES at pH 7.40 and the pipette resistance was between 3 and 5.5 MΩ. Currents were recorded using an Adams & List EPC7 amplifier at 24 h after transfection using Jetprime transfection reagent according to the protocol supplied by Polyplus.

## Results

### Refined multiple sequence alignment and phylogenetic tree for SLC1

To generate homology models of ASCT2, we rely on the availability of experimentally determined structures of its homologs. The accuracy of the homology model highly correlates with the quality of the target-template alignment (Forrest et al., 2006). Previously published models of human SLC1 members were based on Glt_Ph_ which shares a sequence identity of ~30%, where alignments become challenging and more error-prone (Martí-Renom et al., 2000; Eramian et al., 2008). The recent structures of EAAT1, which shares a sequence identity of 46% with ASCT2, provide better templates to model ASCT2, thereby increasing the confidence in the target-template alignment and ASCT2 model. Key structural differences between EAAT1 and Glt_Ph_ include the residue composition of the loop connecting hairpin (HP) 2 and TM8, which is six residues shorter in EAAT1 than in Glt_Ph_ and is more conserved among the human SLC1 members (Canul-Tec et al., 2017) (Supplementary Figure 1). In addition, the N-terminus of TM1 in ASCT2 is more conserved with EAAT1, whose helical structure is nearly parallel to the membrane as opposed to the corresponding loop region in Glt_Ph_. Thus, ASCT2's binding site in the models based on the different proteins are expected to differ in their shape and biophysical properties which in turn will determine the chemical structures of the small molecules recognized by ASCT2 in each conformation, as shown for other SLC transporters (Schlessinger et al., 2012).

**Figure 1:**
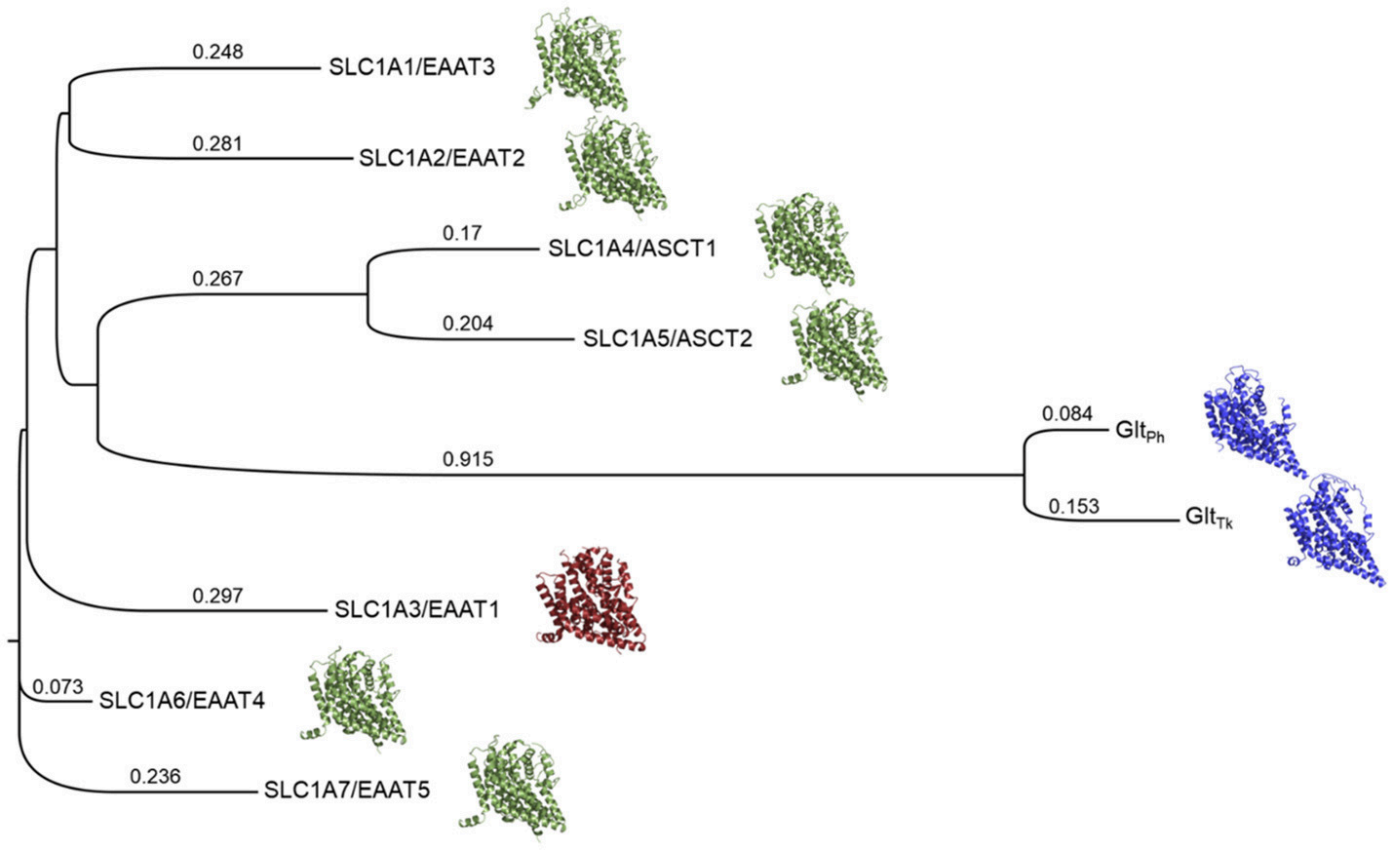
Phylogenetic tree of the human SLC1 transporters and prokaryotic homologs. The solved structures of prokaryotic homologs are depicted in blue; SLC1A3 (EAAT1), the only experimentally determined human member of the SLC1 family, is shown in red. Homology models of the other human SLC1 members are green. Branch lengths and their values correspond to the ratio between the number of substitutions and the alignment length and are proportional to the evolutionary change.

We therefore generated a Multiple Sequence Alignment (MSA) that included the sequences of the human SLC1 family members, as well as the structures of EAAT1 and Glt_Ph_, with Promals3D, which uses structure-based constraints from the alignments of three-dimensional structures (Pei and Grishin, 2014) (Supplementary Figure 1). We then built a phylogenetic tree based on this alignment with Simple Phylogeny (Larkin et al., 2007; McWilliam et al., 2013) (Figure 1). The phylogenetic tree is separated into four distinct branches. The neutral amino acid transporters ASCT1 and ASCT2 cluster together and share sequence identity of 57%, the highest sequence identity among pairs of the SLC1 family. The five glutamate transporters share 44–55% sequence identity, in agreement with previous studies (Kanai et al., 2013). EAAT2 and EAAT3 share the same branch and the highest similarity among the five glutamate transporters.

We then used the MSA to derive pairwise alignments between EAAT1 and each one of the family members, which were used as input for homology modeling (section Materials and Methods) (Figure 1). Interestingly, the models' scores, calculated with Z-DOPE, correlate with the positions of the SLC1 family members on the phylogenetic tree with respect to EAAT1. For example, the EAAT4 model had the best Z-DOPE score (−0.73), whereas the EAAT3 model scored the worst (0.23). These differences likely result from a large insertion between TM4b and TM4c in the EAAT3 (residues 164–201) compared to EAAT1.

### ASCT2 homology models in two different conformations

We generated ASCT2 homology models using MODELLER (Webb and Sali, 2017), based on the EAAT1 structures in the ligand bound outward-open and outward-occluded conformations (section Materials and Methods). The ASCT2 models obtained Z-DOPE scores of −0.31 and −0.52, respectively. These scores were significantly better than those of our previous models based on Glt_Ph_, and were indicative that the models are sufficiently accurate for further analysis (Eramian et al., 2008; Colas et al., 2015). Initial models were refined by iteratively modeling the sidechains on a fixed backbone of various binding site residues with PyMOL and visually assessing the optimized models (section Materials and Methods).

**Figure 2:**
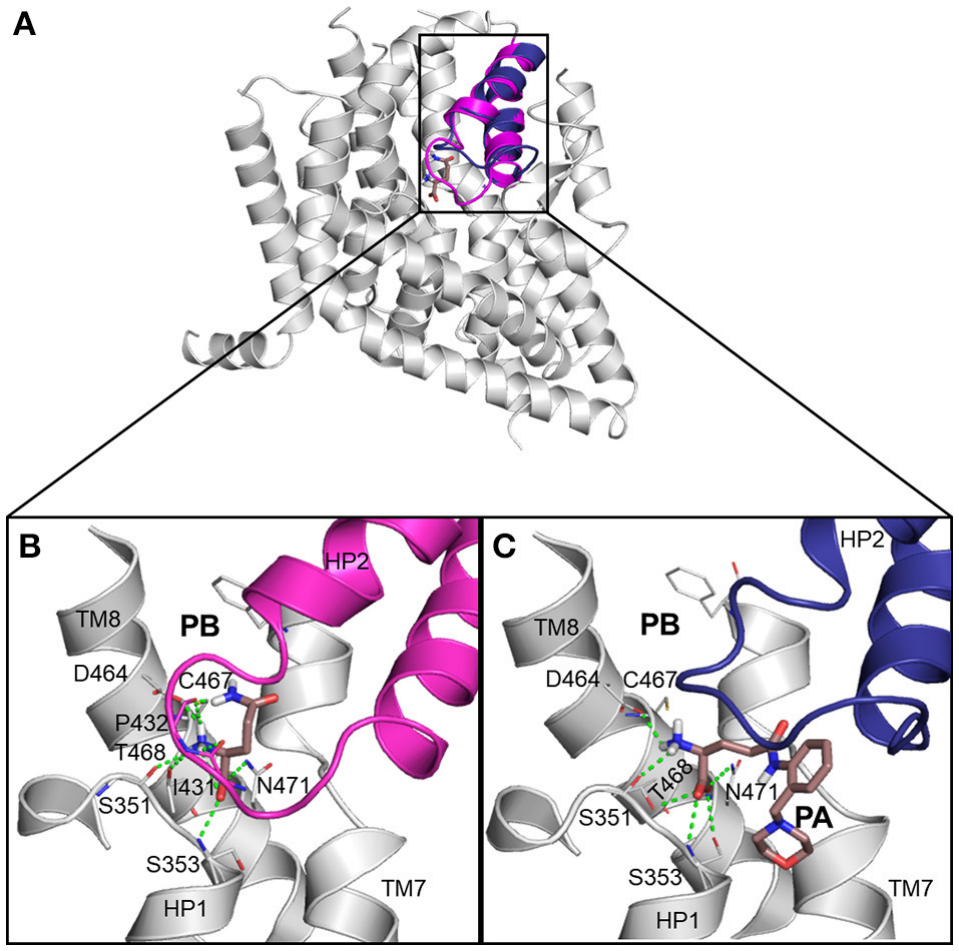
ASCT2 models in two conformations. **(A)** The outward-occluded and outward-open models and corresponding predicted binding modes for known ligands of ASCT2, including **(B)** a substrate and **(C)** inhibitor. HP2 is highlighted with magenta and blue ribbons. The sidechain atoms of key residues are illustrated with gray lines and the small molecule ligands are displayed as salmon sticks, with oxygen, nitrogen, and sulfur atoms in red, blue, and yellow, respectively. Hydrogen bonds between the binding site residues I431, P432, S351, S353, D464, T468, and N471, and the ligand are represented by dashed green lines. **(B)** Outward-occluded model with docked glutamine. **(C)** Outward-open model shown with (2S)-2-amino-4({2-[(morpholin-4-yl)methyl]phenyl}carbamoyl)butanoic acid. HP2 is in an outward-open conformation.

Next, we docked known ASCT2 ligands, including substrates and inhibitors to the substrate binding site of the models in both conformations. Notably, the mode of ligand binding in the outward-occluded model, closely resembles that of the outward-open model, recapitulating the ligand poses in the crystal structures of EAAT1 and Glt_Ph_ (Figures 2B,C). Critical polar interactions between the amino-acid like ligands and the binding site are mostly conserved between the two conformations. For example, in the outward-occluded conformation (Figure 2B), the carboxy group of the substrate glutamine forms hydrogen bonds with S353 (HP1), as well as with T468 and N471 (TM8); and the amino group of the substrate forms hydrogen bonds with S351 (HP1) and I431 (HP2) and D464 and T468 (TM8). However, in the outward-open conformation, with a docked inhibitor, there are no contacts with HP2 (Figure 2C).

It has been shown that C467 in ASCT2, which corresponds to R479 and T459 in EAAT1 and ASCT1, respectively, plays a key role in substrate specificity (Scopelliti et al., 2013, 2018; Colas et al., 2015). Mutating this residue to either a neutral amino acid or an arginine, can alter substrate preference in the SLC1 transporters (Conradt and Stoffel, 1995; Scopelliti et al., 2013). For example, the T459R mutation in ASCT1 introduces acidic amino acid transport to the neutral amino acid transporter (Scopelliti et al., 2013). Equally important is this residue's role in facilitating the availability of a hydrophobic subpocket termed pocket B (PB), which increases the size and changes its shape of the binding site (Figures 2, **4**). In the outward-open conformation, there is an additional subpocket, pocket A (PA) that is revealed when HP2 adopts an open conformation. The residues constituting PA are variable among Glt_Ph_-R397C, EAAT1, and our model of ASCT2 (Table 1). One potential strategy to identify chemically novel ASCT2 inhibitors is by targeting both subpockets PA and PB simultaneously.

**Table 1:** Pocket A residues and their respective protein locations aligned between ASCT2 and EAAT1 and Glt_Ph_-R397C.

**Location**	**ASCT2**	**EAAT1**	**Glt_*Ph*_-R397C**
	**Residue**	**Residue**	**Residue**
TM2		V96I^(a)^	V58
		I100	
TM4C		A243I^(b)^	V151
HP1	S352	S364	S277
	S353	S365	S278
	S354	S366	S279
	A355	A367	G280
	L357	L369	L282
TM7	P380	P392	
		V393	
	A383	A395	A307
	T384	T396	T308
		I397	I309
		N398	
HP2	S425	S437	
	V426	I438	
	G427	G439	
	A428	A440	T352
	A429	A441	A353
	G430	G442	G354
	I431	I443	V355
	P432	P444	P356
	A433		G357
	G434		A358

(a) V96 and

(b) *A234 is mutated to I in EAAT1 crystal (5MJU)*.

### Comparison between SLC1 structures and models

**Figure 3:**
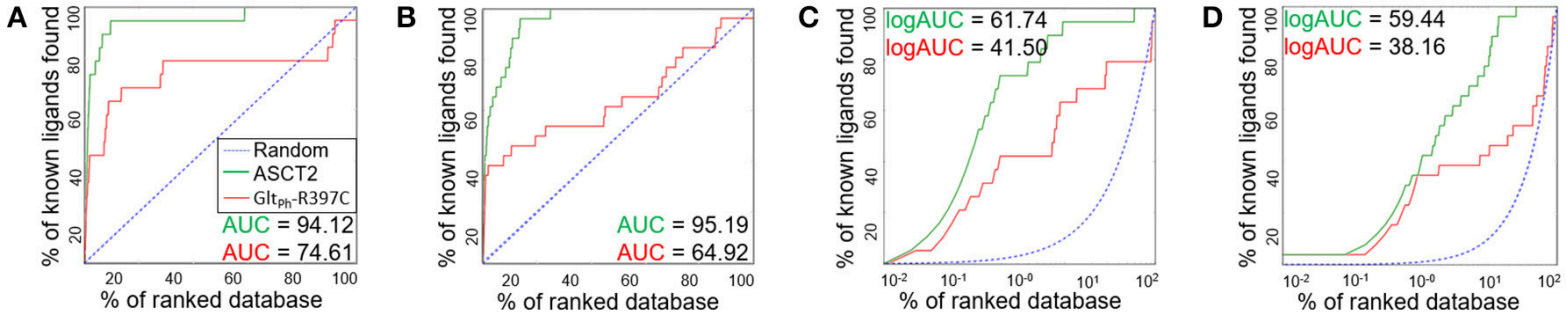
Model Evaluation with enrichment plots. Enrichment plots are shown for the ASCT2 models (green) and Glt_Ph_-R397C variants (red) in two conformations; the plot that is expected by a random selection of ligands is represented by a blue dashed line. The enrichment plots are shown for the outward-occluded conformation (**A,C**) and outward-open conformation (**B,D**). with the area under the curve (AUC) (**A,B**) and the logarithmic AUC (logAUC) (**C,D**) calculated for each plot.

We assessed the relevance of the ASCT2 models for structure-based ligand discovery. ASCT2 models in both conformations were evaluated for their ability to distinguish ligands from likely-non-binders or decoys using docking (“ligand enrichment”) (Huang et al., 2006; Fan et al., 2009). Here, the decoys are molecules that resemble the known ligands physically but are topologically dissimilar to minimize the likelihood of binding (Mysinger et al., 2012). We calculated the area under the curve (AUC) of enrichment plots as well as the logarithmic AUC (logAUC), which determines the model's ability to prioritize those known ligands early in the library. The AUC of the outward-occluded and outward-open models were 94.12 and 95.19, respectively (Figures 3A,B), suggesting that both models are relevant for ligand discovery with molecular docking (Fan et al., 2009; Schlessinger et al., 2011; Colas et al., 2015).

We also calculated the AUC and logAUC for the Glt_Ph_ variant structures (Glt_Ph_-R397C) that were engineered to mimic ASCT2 function (Scopelliti et al., 2018). In brief, the Glt_Ph_-R397C variant has a point mutation in the binding site residue R397C, which corresponds to C467 in ASCT2, allowing this transporter to bind and transport substrates of ASCT2 such as serine and alanine. Indeed, the Glt_Ph_-R397C outward-occluded and outward-open structures obtained AUCs of 74.61 and 64.92, respectively, suggesting that they capture known ASCT2 ligands (Figures 3A,B); however, the enrichment scores are lower than those obtained using the new ASCT2 models.

**Figure 4:**
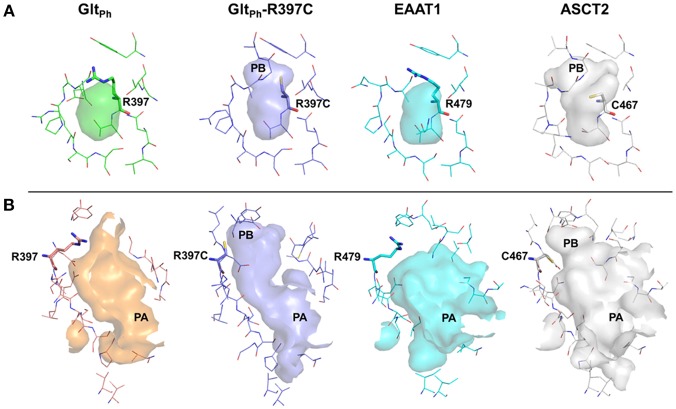
The substrate binding site of ASCT2 and its homologs. The binding site is compared between Glt_Ph_ (green) Glt_Ph_-R397C (purple), EAAT1 (cyan), and ASCT2 (gray) in the outward-occluded and open conformations. Surface representations of the substrate binding site are shown, highlighting PA and PB. Key residues R397 (Glt_Ph_) R397C (Glt_Ph_-R397C), R479 (EAAT1) and C467 (ASCT2) are depicted as sticks. **(A)** Outward-occluded conformation, **(B)** outward-open conformation rotated 180° degrees to view PA.

To rationalize the difference in enrichment scores and in ligand specificity, we analyzed the binding sites of our ASCT2 models, and the EAAT1 and Glt_Ph_-R397C structures. In both wildtype Glt_Ph_ [PDB codes: 6BAT (Scopelliti et al., 2018), 2NWW (Yernool et al., 2004)] and EAAT1 (Canul-Tec et al., 2017), PB is blocked by R397/R479 in both the outward-occluded and outward-open conformations (Figure 4A). Particularly, the binding site of Glt_Ph_-R397C occluded (6BAU) is almost identical to the binding site of the corresponding conformation of the wild-type Glt_Ph_ (6BAT) (RMSD 0.24).

**Figure 5:**
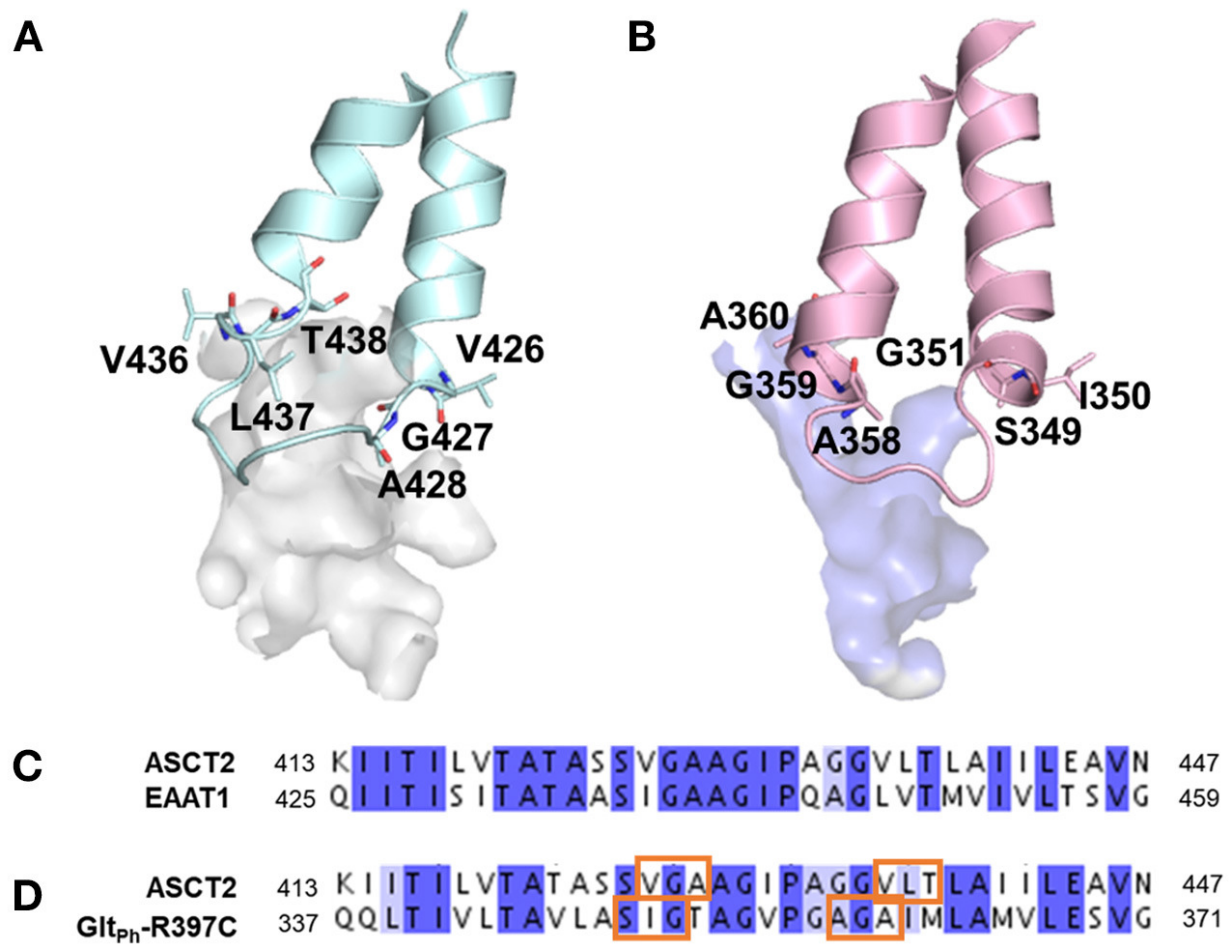
Differences in pocket size and shape in the outward-open conformation. The HP2 is shown in blue and pink for **(A)** ASCT2, **(B)** Glt_Ph_-R397C, respectively. Key residues are illustrated with sticks and oxygen and nitrogen, and sulfur atoms in red, and blue. Sequence alignment of HP2 **(C)** between ASCT2 and EAAT1 and **(D)** between ASCT2 and Glt_Ph_-R397C. Orange boxes mark the residues that differ in secondary structure between ASCT2 and Glt_Ph_-R397C.

Interestingly, larger inhibitors are ranked earlier in library for the ASCT2 models than in the Glt_Ph_-R397C structures. This can be explained by the difference in the size of the binding sites of the two proteins. The ASCT2 substrate binding site in both conformations is larger (outward-occluded: 70 Å^3^, outward-open: 439 Å^3^) than those of Glt_Ph_-R397C (45Å^3^, 283 Å^3^). In addition, a striking difference between the two proteins is observed in PA, which is divergent in sequence and structure between ASCT2 and Glt_Ph_-R397C (Figures 4B, Figure 5 and Table 1). Particularly, the HP2 residues in ASCT2 V426, G427, A428, and V436, L437, T438 form a loop region, while the corresponding residues in Glt_Ph_-R397C form a helical structure (Figures 5A,B). HP2 is more conserved between ASCT2 and EAAT1 than between ASCT2 and Glt_Ph_-R397C (Figures 5C,D). These results indicate that while this Glt_Ph_-R397C variant is useful to describe substrate specificity, it may not capture all the binding site features that are relevant for ASCT2-inhibitor interactions (Figure 2).

### Discovery of a new ASCT2 inhibitor

**Figure 6:**
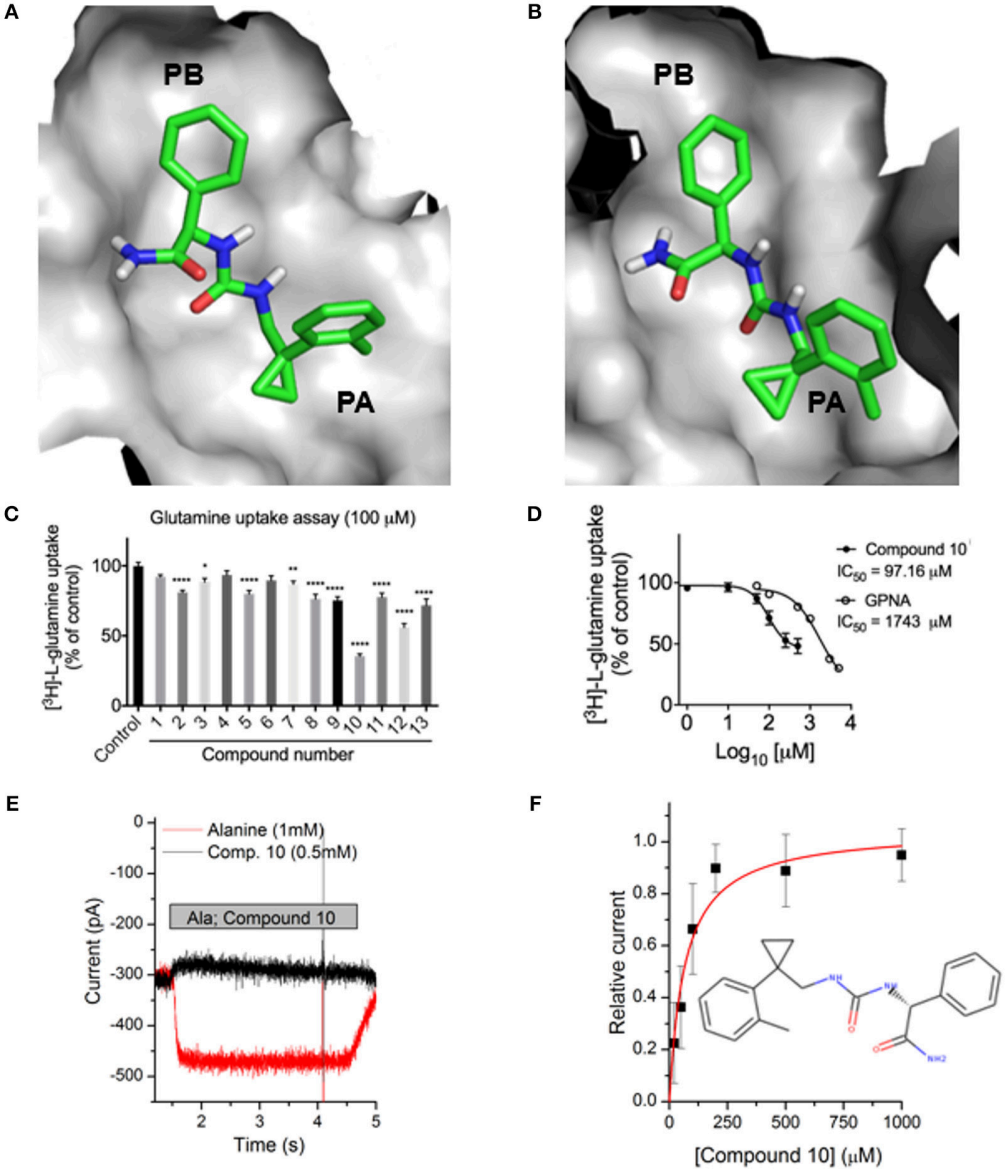
Identification of a novel ASCT2 inhibitor. Predicted mode of binding of compound **10** in **(A)** ASCT2 models based on Glt_Ph_ and **(B)** ASCT2 models based on EAAT1. **(C)** Uptake of [^3^H]-L-glutamine in SK-MEL-28 cells incubated in the presence and absence of 13 small molecules. Uptake of [^3^H]-L-glutamine was assessed over 15 minutes and compared to DMSO vehicle control. Data are the mean ± standard error of the mean of 3-4 experiments per compound. Significance was assessed using a one-way ANOVA with multiple comparisons (GraphPad Prism), where *P < 0.05, **P < 0.01, and ****P < 0.0001. **(D)** IC_50_ calculation SK-MEL-28 cells were incubated in the presence of compound **10** or GPNA at a range of concentrations. Uptake of [^3^H]-L-glutamine was assessed over 15 min, log transformed and IC_50_ determined using a Non-linear fit (log[inhibitor] vs. response, variable slope, four parameters; GraphPad Prism). Data are the mean ± standard error of the mean of 3-5 experiments per concentration. **(E)** Electrophysiological characterization of inhibitory activity on ASCT2. Representative original currents induced by application (indicated by gray bar) of 1 mM alanine (red) and 0.5 mM compound **10** (black). The internal solution contained 130 mM NaSCN and 10 mM alanine, the external solution 140 mM NaCl. **(F)** Dose response curve for compound **10**, the red line is a fit to a one-site binding equation with an IC_50_ of 67 ± 17 μM.

Previously identified ASCT2 ligands are predicted to bind only one of the two subpockets PA and PB (Colas et al., 2015; Singh et al., 2017). We hypothesized that targeting both PA and PB simultaneously with virtual screening may yield compounds with chemically novel scaffolds for ASCT2. In parallel to our ASCT2 modeling based on the EAAT1 structure, we conducted a discovery campaign using our previously published Glt_Ph_-based ASCT2 models. Our rationale was to use these compounds to iteratively refine the outward EAAT1-based ASCT2 models. The lead-like library from ZINC15 was docked against the Glt_Ph_-based ASCT2 models (section Materials and Methods). Compounds were prioritized on the basis of chemotype novelty and predicted mode of binding to PA and PB. Thirteen compounds were tested for activity using glutamine uptake assays in a melanoma cell line (SK-MEL-28). Three compounds significantly inhibited [^3^H]-L-glutamine uptake in SK-MEL-28 cells at 100 μM (Figure 6C, Supplementary Figure 2). We next determined the IC_50_ of the most potent compound, compound **10** (ZINC69811181) for inhibition of glutamine uptake in SK-MEL-28 cells, using a range of concentrations. We observed an IC_50_ of 97.16 μM for compound **10** (Figure 6D), which is ~18-fold better than the ASCT2 inhibitor L-γ-glutamyl-p-nitroanilide, our positive control (GPNA; IC_50_ = 1743 μM; Figure 6D). The compound was docked to our new outward-open model of ASCT2 and is predicted to have a similar mode of binding to that proposed in our previous model (Figures 6A,B). Compound **10** was further analyzed using electrophysiology (Figure 6E) to confirm target engagement with ASCT2. Application of compound **10** to ASCT2-expressing HEK293 cells resulted in inhibition of the anion leak current, which was previously shown to be the hallmark of ASCT2 inhibitors (Albers et al., 2012; Colas et al., 2015; Singh et al., 2017). In contrast, transported substrates, like alanine, induce inward current in the presence of intracellular anion, due to increased anion efflux (Figure 6E). The effect of compound **10** was dose dependent, saturating with an IC_50_ of 67 ± 17 μM, in the same range of that found using the glutamine uptake assay (Figure 6F). Compound **10** provides a useful scaffold for further optimization and proof-of-concept for the design of more selective and higher affinity compounds for ASCT2.

## Discussion

The discovery of glutamine addicted tumors has proposed that limiting glutamine to cancer cells results in malignant cell death (Akyuz et al., 2013). One approach to target glutamine addicted tumors is to inhibit glutamine import into the cancer cells through nutrient transporters. ASCT2 plays a key role in glutamine transport in multiple cancers (Wasa et al., 1996; Collins et al., 1998; Fuchs et al., 2007) and as a result it is now seen as a potential anticancer drug target. Currently, there are no drugs in the clinic that have been specifically designed to target nutrient transporters implicated in tumorigenesis (César-Razquin et al., 2015). One challenge in studying ASCT2, is the lack of an experimentally determined structure. In structure-based ligand discovery, a detailed understanding of the substrate binding site is needed to develop tool compounds. These compounds will enable the further study and development of future drugs for ASCT2. For this reason, we have modeled ASCT2 with a new alignment and template structures and investigated the potential utility of these models in small molecule discovery. Three major findings emerge in this work. First, we generated a refined, new multiple sequence alignment for the SLC1 family, inclusive of its prokaryotic homologs and experimentally determined structures, to be used in the modeling of the SLC1 family members. The accuracy of the target-template alignment is critical to the generation of an accurate homology models (Fiser and Sali, 2003; Forrest et al., 2006). We hypothesized that the inclusion of structural information from the newly solved EAAT1 structures would improve the alignment and therefore our models (Supplementary Figure 1). Indeed, the new models obtained better Z-DOPE scores and enrichment scores than those of previously reported ASCT2 models (Colas et al., 2015). This new alignment provides a useful resource for others to build homology models for the SLC1 family. Additionally, the initial models generated in this study are available for direct download (section Materials and Methods).

The second key result of this study is that the newly-generated ASCT2 models accurately approximate inhibitor binding, as measured by their ability to distinguish between known ligands vs likely-non-binders with docking. The models enriched better than X-ray structures of Glt_Ph_ variant structures (Glt_Ph_-R397C) that mimic ASCT2 and twice as well as the previously published ASCT2 models by our lab (Colas et al., 2015). While our previous models have shown utility for the discovery of unique ASCT2 inhibitors, the new models are of higher resolution, providing the foundation for the rational design of potent inhibitors for ASCT2. Closer examination of the binding sites of the various SLC1 members, including EAAT1, ASCT2, and Glt_Ph_, suggests differences in several residues that affect the size and shape of pocket A (PA) (Figures 2, 4). This information may inform the design of conformation-specific ASCT2 small molecule ligands.

Third, we identified an ASCT2 inhibitor with a unique predicted mode of binding. One key challenge in the development of tool compounds for ASCT2 is to discover ligands that deviate from amino acid structures, and therefore may compete better with the high levels of circulating alanine and glutamine (~450–550 μM) (Cantor et al., 2017). Such molecules are expected to have improved pharmacokinetic properties and ASCT2 specificity. For example, compounds inhibiting EAAT1 may have various deleterious neurological effects. Our computational screen followed by experimental testing, identified compound **10**, a weak inhibitor of ASCT2 that does not contain amino-acid like structure. Interestingly, this compound is predicted to bind to PA, which is different in size and shape in the EAATs due to dissimilarity in HP2 and helix packing. This compound also binds PB, which has subtle structural differences among the human SLC1 members. Notably, compound **10** is 18-fold more potent than GPNA, which is also predicted to bind PA; thus, this compound provides a potential starting point for optimization in future drug discovery campaigns targeting ASCT2.

## Author contributions

Homology modeling, molecular docking, and analysis was conducted by RAG with guidance by AS. Virtual screening of ZINC library was done by CC with guidance by AS. Experimental design and testing was performed by NO and JH. Additional analysis was conducted by CG. All authors contributed to the preparation of the manuscript.

### Conflict of interest statement

The authors declare that the research was conducted in the absence of any commercial or financial relationships that could be construed as a potential conflict of interest.

## Footnotes

^1^The PyMOL Molecular Graphics System, V.S., Llc.

## Abbreviations

SLC Transporters: Solute Carrier Transporters
SLC1: Solute Carrier Family 1
RMSD: Root mean square deviation
MSA: multiple sequence alignment
ASCT2: alanine-serine-cysteine transporter 2
EAAT: excitatory amino acid transporter
DOPE score: Discrete Optimized Protein Energy.
