# Supplementary material for: Homology Modeling Informs Ligand Discovery for the Glutamine Transporter ASCT2
Source: Front Chem. 2018 Jul 24;6:279. doi: 10.3389/fchem.2018.00279 (PMC6066518; doi:10.3389/fchem.2018.00279)
Supplement: Supplementary file 1 [file Table_1.DOCX]

**Table 1 – Source of screening compounds used in uptake assays**

| **Drug #** | **ZINCID** | **Company** | **Catalogue #** | **Structure** |
| --- | --- | --- | --- | --- |
| 1 | 10434760 | Chembridge | 9079483 | 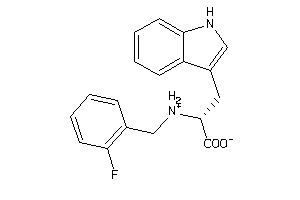 |
| 2 | 96163354 | Chembridge | 80158431 | 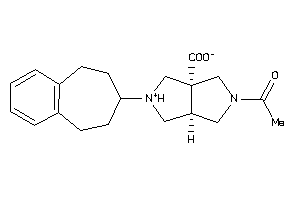 |
| 3 | 10475472 | Chembridge | 9152774 | 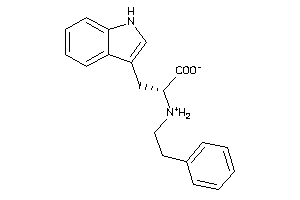 |
| 4 | 10475482 | Chembridge | 9149496 | 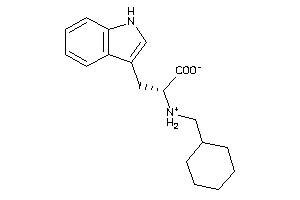 |
| 5 | 92028709 | Chembridge | 92463109 | 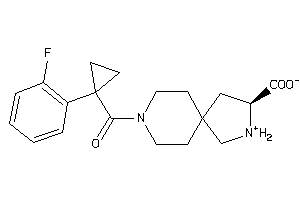 |
| 6 | 2512908 | Sigma-Aldrich | CDS010289 | 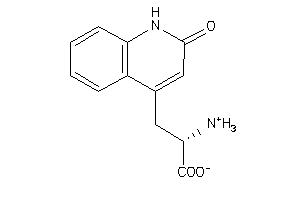 |
| 7 | 402754 | Combi-Blocks | MolPort-019-923-034  Combi-Blocks: QB-1649 | 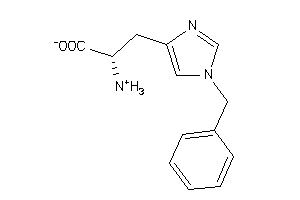 |
| 8 | 2117715 | Vitas-M Laboratory, Ltd. | MolPort-019-913-510  STL129478 | 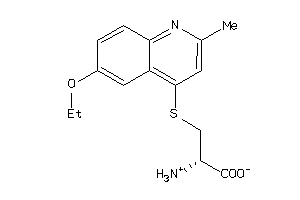 |
| 9 | 48888816 | Life Chemicals | F6252-8238 | 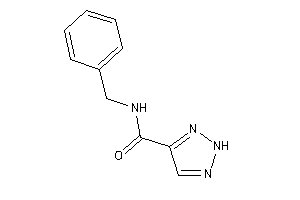 |
| 10 | 69811181 | Enamine | Z1028119242 | 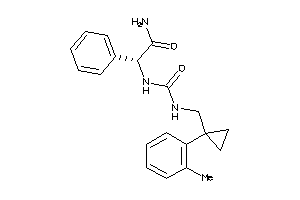 |
| 11 | 89575628 | Enamine | Z1444221622 | 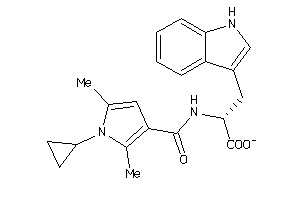 |
| 12 | 69707916 | Enamine | Z1171352641 | 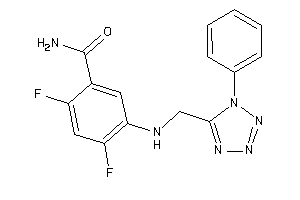 |
| 13 | 19427538 | Enamine | Z1336457514 | 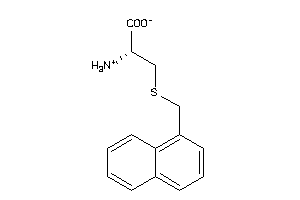 |
