# Supplementary material for: Homology Modeling Informs Ligand Discovery for the Glutamine Transporter ASCT2
Source: Front Chem. 2018 Jul 24;6:279. doi: 10.3389/fchem.2018.00279 (PMC6066518; doi:10.3389/fchem.2018.00279)
Supplement: Supplementary file 2 [file Image_1.pdf]

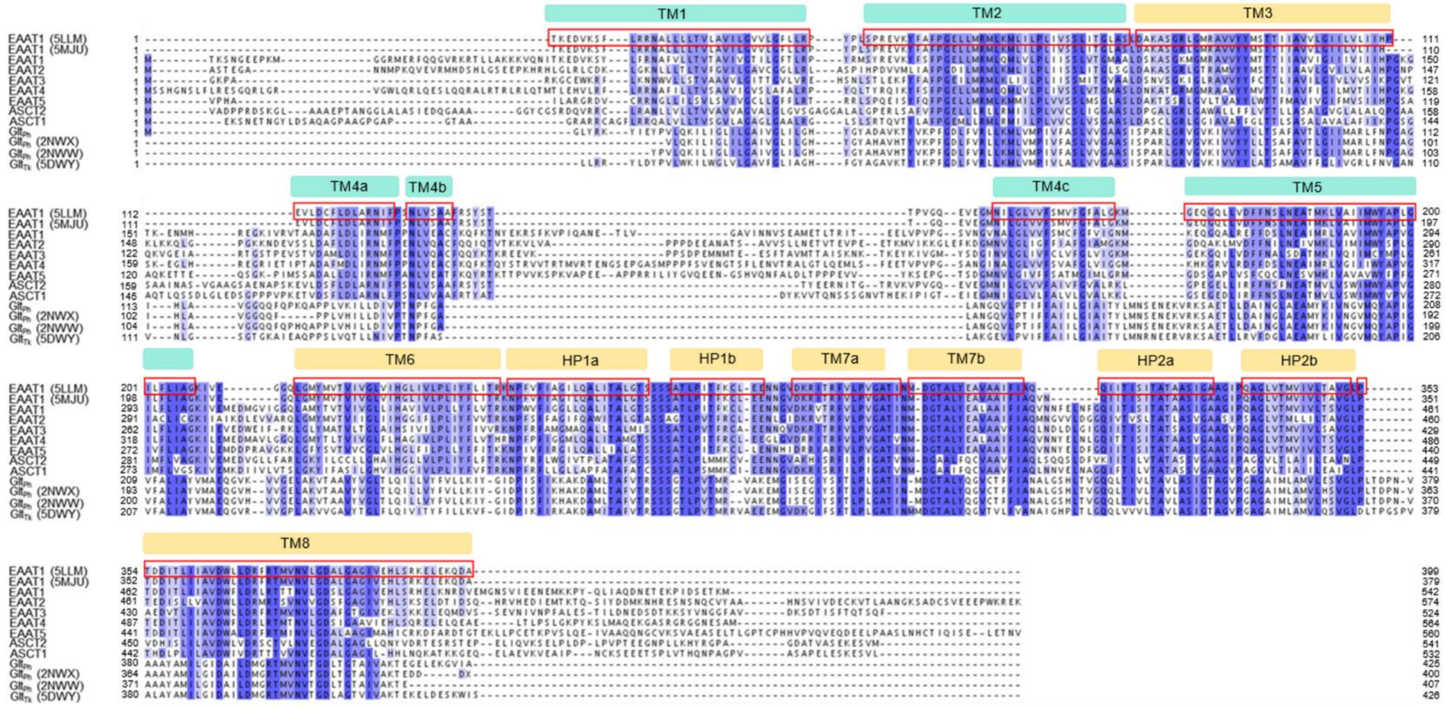

Supplementary Figure 1. Multiple sequence alignment of the SLC1 family and prokaryotic homologs**.** Promals3D alignment visualized in JalView highlighting conserved residues in blue. TM regions denoted according to the EAAT1 structure are marked by red boxes. TM helices involved in the mobile transport domain are highlighted in mint green, TM helices comprising the scaffold domain are shown in yellow.
