# Supplementary material for: Homology Modeling Informs Ligand Discovery for the Glutamine Transporter ASCT2
Source: Front Chem. 2018 Jul 24;6:279. doi: 10.3389/fchem.2018.00279 (PMC6066518; doi:10.3389/fchem.2018.00279)
Supplement: Supplementary file 3 [file Image_2.PDF]

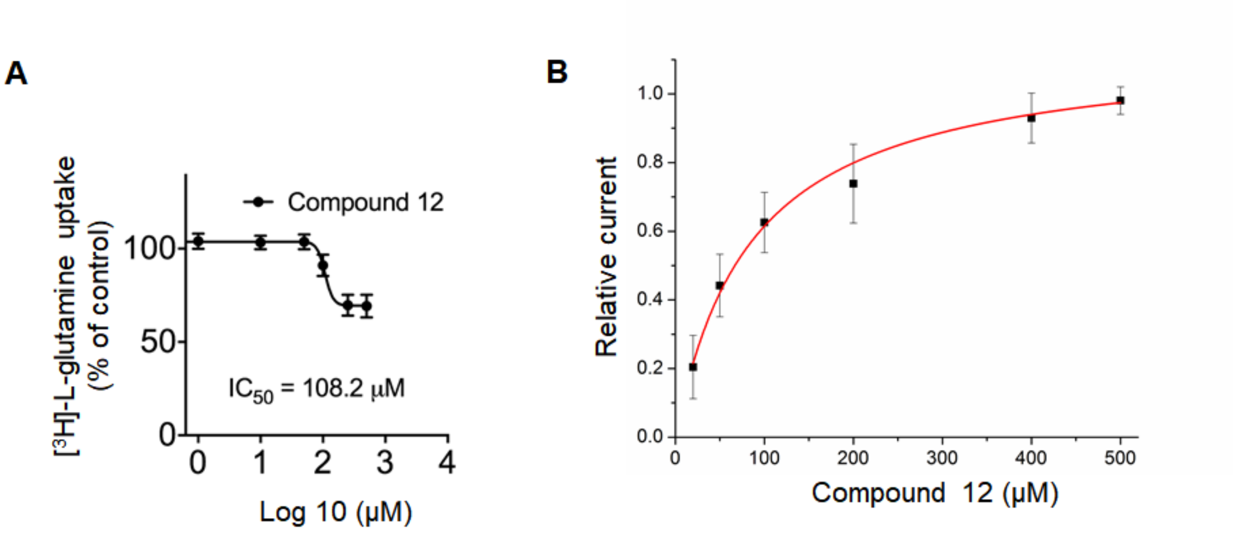

Supplementary Figure 2. (A) IC_{50} calculation SK-MEL-28 cells were incubated in the presence of Compound **12** (ZINC ID 69707916) at a range of concentrations. Uptake of [^{3}H]-L-glutamine was assessed over 15 minutes, log transformed and IC_{50} determined using a Non-linear fit (log[inhibitor] vs response, variable slope, four parameters; GraphPad Prism). Data are the mean +/- standard error of the mean of 4 experiments per concentration. **(B)** Dose response curve for compound **12**, the red line is a fit to a one-site binding equation with an IC50 of 108 µM. The internal solution contained 130 mM NaSCN and 10 mM alanine, the external solution 140 mM NaCl (Methods).
